# Comparative Transcriptomic Analysis Reveals Domestication and Improvement Patterns of Broomcorn Millet (*Panicum miliaceum* L.)

**DOI:** 10.3390/ijms252011012

**Published:** 2024-10-13

**Authors:** Xinyu Zhao, Minxuan Liu, Chunxiang Li, Jingyi Zhang, Tianshu Li, Fengjie Sun, Ping Lu, Yue Xu

**Affiliations:** 1 School of Life Sciences, Jilin University, Changchun 130012, China; xinyuzhao498@gmail.com (X.Z.); chunxiangli@jlu.edu.cn (C.L.); zhangjingyi2001@yeah.net (J.Z.); lts@jlu.edu.cn (T.L.); 2 School of Life Sciences, Northeast Normal University, Changchun 130021, China; 3 Institute of Crop Science, Chinese Academy of Agricultural Sciences, Beijing 100081, China; liuminxuan@caas.cn (M.L.); luping@caas.cn (P.L.); 4 Department of Biological Sciences, School of Science and Technology, Georgia Gwinnett College, Lawrenceville, GA 30043, USA; fsun@ggc.edu; 5 National and Local United Engineering Laboratory for Chinese Herbal Medicine Breeding and Cultivation, School of Life Sciences, Jilin University, Changchun 130012, China

**Keywords:** seedling and filling stages, differentially expressed gene, reproductive and vegetative traits

## Abstract

Broomcorn millet (*Panicum miliaceum* L.) is one of the earliest crops, domesticated nearly 8000 years ago in northern China. It gradually spread across the entire Eurasian continent, as well as to America and Africa, with recent improvement in various reproductive and vegetative traits. To identify the genes that were selected during the domestication and improvement processes, we performed a comparative transcriptome analysis based on wild types, landraces, and improved cultivars of broomcorn millet at both seeding and filling stages. The variations in gene expression patterns between wild types and landraces and between landraces and improved cultivars were further evaluated to explore the molecular mechanisms underlying the domestication and improvement of broomcorn millet. A total of 2155 and 3033 candidate genes involved in domestication and a total of 84 and 180 candidate genes related to improvement were identified at seedling and filling stages of broomcorn millet, respectively. The annotation results suggested that the genes related to metabolites, stress resistance, and plant hormones were widely selected during both domestication and improvement processes, while some genes were exclusively selected in either domestication or improvement stages, with higher selection pressure detected in the domestication process. Furthermore, some domestication- and improvement-related genes involved in stress resistance either lost their functions or reduced their expression levels due to the trade-offs between stress resistance and productivity. This study provided novel genetic materials for further molecular breeding of broomcorn millet varieties with improved agronomic traits.

## 1. Introduction

Domestication is generally considered the first stage of plant breeding and cultivation [1]. After a long period of domestication, crops are usually characterized by a variety of enhanced traits, e.g., a loss of seed shattering [2], decreased tiller number [3,4], enlarged fruit [5], loss of seed dormancy [6], upright and strong stem [7,8], and increased seed size [9], due to both artificial and natural selection. These characteristics are usually referred to as ‘domestication syndrome’. In general, the variations of these morphological characteristics between wild relatives and landraces (i.e., traditional varieties of a species that have developed over time through natural selection and local agricultural practices) are significantly greater than those within either wild relatives or landraces [10]. Additionally, the initial domestication stage of crops is frequently followed by a second stage of improvement from landraces to improved cultivars, with yield, color, flavor, and other selected agronomic traits [11,12]. There is an increasing number of reports performing comparative genomic or transcriptomic analyses between wild types and landraces or between landraces and improved cultivars of crops to identify genes related to domestication or improvement processes in various crop plants, such as broomcorn millet [13,14], rice [15,16,17], maize [18,19,20], soybean [21,22,23,24], wheat [25], foxtail millet [26], sorghum [27], common bean [9,28], tartary buckwheat [29], sea-island cotton [30], tomato [31,32], melon [33], water caltrop [34], pepper [35], cauliflower [36], carrot [37], cowpea [38], mulberry [39], and sunflower [40].

Broomcorn millet (*Panicum miliaceum* L.) is one of the earliest crops domesticated in the world, showing the characteristics of a short growing season and high tolerance to heat and drought [41]. It is well known that broomcorn millet was first domesticated in northern China at least 8000 years ago and then spread to the entire Eurasia continent, America, and Africa [42,43,44,45,46,47,48,49,50,51]. Although it is commonly accepted that the weedy broomcorn millet [*Panicum ruderale* (Kitag.) Chang or *Panicum miliaceum* subsp. *ruderale* (Kitag.) Tzvel], widely distributed in Eurasia and America, is considered the descendant of the wild ancestor of cultivated broomcorn millet [14,41,47,52,53], while some individuals of the feral type derived from either reverse mutations of cultivars or introgressions between cultivars and their wild relatives are detected in populations of weedy broomcorn millet [47,53]. The main differences in morphological characteristics between weedy and cultivated broomcorn millet include pericarp color (dark gray or dark brown vs. white, yellow, brown, or red), plant height (40–100 vs. 100–150 cm), panicle type (loose vs. close, loose, or lateral), tiller number (4–10 vs. 1–3), seed color (dark yellow vs. yellow or light yellow), and seed size (small vs. large) [41,54,55]. Additionally, a genetic analysis of broomcorn millets suggested that weedy accessions contained many beneficial variations that were largely lost in cultivated accessions [14]. However, there are no significant differences in morphological characteristics between landraces and improved cultivars due to a relatively short improvement history of broomcorn millet in China, which began in the 1940s [41], while some traits (such as plant height, seed number, seed size, seed shattering, and plant lodging) related to the agricultural production of improved cultivars are much more optimized than those of the landraces. Similarly, compared with landraces, improved cultivars have also shown enhanced taste, nutrition, and resistance to drought, salt, shoot fly, and smut [41,56]. Therefore, the studies of genes and related metabolic pathways involved in the domestication and improvement processes of these production-, taste-, nutrition-, and stress resistance-related characteristics are of significant importance in the research of broomcorn millet. These studies could make a great impact on the molecular breeding of broomcorn millet with improved agronomic traits. A previous resequencing study of 516 broomcorn millet accessions of wild types, landraces, and improved cultivars identified a total of 139 loci associated with 31 key domestication and agronomic traits based on sequence diversity [13]. Furthermore, Lu et al. constructed a comprehensive variation map of both weedy and cultivated broomcorn millet based on a large-scale genomic sequencing of 1904 weedy and cultivated accessions, and they identified a total of 186 loci for 12 agronomic traits [14]. Additionally, Liu et al. established an *Agrobacterium tumefaciens*-mediated genetic transformation protocol and a clustered regularly interspaced short palindromic repeats (CRISPR)/Cas9-mediated genome-editing system based on broomcorn millet [57], providing great potential for further exploration and application of domestication- and improvement-related genes in molecular breeding and the improvement of broomcorn millet. However, few studies have been reported on genes related to the domestication and improvement of broomcorn millet, specifically based on gene expression diversity, e.g., a differential gene expression analysis was performed between eight wild type and twenty-four cultivated accessions of broomcorn millet to identify the PAV genes enriched in resistance-related genomic regions [13]. Further systematic studies are still needed to identify DEGs associated specifically with the domestication and improvement of broomcorn millet.

RNA sequencing (RNA-Seq) is a high-throughput technology commonly used for comprehensive transcriptome analysis [58]. It has been frequently applied to identify differentially expressed genes (DEGs), sequence variants (i.e., differences in the nucleotide sequence of DNA or RNA between individuals or populations), novel transcripts (i.e., newly identified RNA sequences that were not previously annotated or characterized in the existing genomic databases), alternative spliced transcripts (i.e., RNA molecules produced from a single gene through a process called alternative splicing, allowing a single gene to generate multiple distinct mRNA transcripts), and gene co-expression networks in various organisms. Up to now, the transcriptome analysis of broomcorn millet was primarily focused on the molecular mechanisms regulating stress resistance, as well as plant growth and development [59,60,61,62,63]. Therefore, it holds significant potential for studying the domestication and improvement processes of broomcorn millet based on a comparative transcriptome analysis, which is essential to enhance our understanding of the molecular mechanisms underlying these processes of broomcorn millet. Furthermore, our previous studies based on microsatellite markers and specific-locus amplified fragment sequencing (SLAF-Seq—a molecular technique used to discover and analyze genetic variation in specific genomic regions, primarily used for identifying single nucleotide polymorphisms and other types of genetic variations in a targeted manner, often in plants or organisms with complex genomes.) showed that the genetic diversity of weedy broomcorn millet is significantly higher than that of cultivated broomcorn millet [47,53]. These studies strongly indicate that enhancing our understanding of the domestication and improvement processes would enable further exploration of elite genetic resources in the weedy broomcorn millet, help overcome the current bottleneck in developing improved varieties caused by limited genetic diversity, and identify novel genetic materials for further molecular breeding of broomcorn millet varieties with improved agronomic traits.

In this study, the transcriptomes of wild types, landraces, and improved cultivars of broomcorn millet at both seedling and filling stages were sequenced by RNA-Seq. The comparative transcriptome analysis was performed between wild types and landraces as well as between landraces and improved cultivars of broomcorn millet at both seedling and filling stages. The goals of our study were as follows: (1) to identify the candidate genes associated with the domestication and improvement processes of broomcorn millet based on a differential gene expression analysis and weighted gene co-expression network analysis (WGCNA); (2) to reveal the metabolic pathways involved in the domestication and improvement processes of broomcorn millet based on functional annotation and enrichment analyses of the DEGs identified among wild types, landraces, and improved cultivars of broomcorn millet at seedling and filling stages; and (3) to further explore the molecular mechanisms underlying the domestication and improvement processes of broomcorn millet, providing novel genetic materials and experimental evidence to support future molecular breeding of broomcorn millet with enhanced agronomic traits.

## 2. Results

### 2.1. Qualitative Evaluation of RNA-Seq

To investigate the evolutionary patterns of the domestication and improvement processes of broomcorn millet, transcriptome analyses were performed based on a total of 36 individual plants (two wild type, two landrace, and two improved cultivar accessions with three individuals per accession; Table S1) at seedling (18 plants) and filling (18 plants) stages using RNA-Seq. After the removal of adapter sequences, ambiguous nucleotides, and low-quality sequences, a total of 236.82 Gb clean data were obtained, with the number of clean reads of each sample ranging from 19,166,201 to 25,803,592 and more than 93.4% of bases in each sample having a Q-score (a measure used in genomics to assess the quality of sequencing data) no less than 30. The clean reads were mapped onto the broomcorn millet genome (https://www.ncbi.nlm.nih.gov/assembly/GCA_003046395.2/; accessed on 25 May 2023), with mapping ratios ranging from 32.65% to 95.68% in these samples, suggesting sufficient data for subsequent analyses (Table S1). The raw RNA-Seq data were deposited at the National Center for Biotechnology Information (NCBI; https://www.ncbi.nlm.nih.gov/bioproject/; accessed on 30 July 2024) database with an accession number of PRJNA1141739.

**Figure 1:**
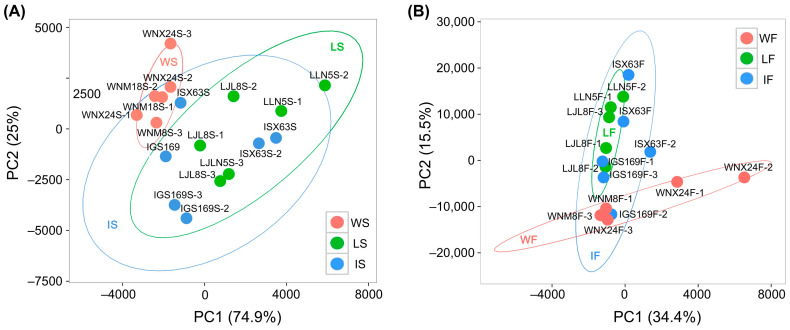
Principal component analysis (PCA) of broomcorn millet samples based on FPKM values of genes. (**A**) PCA plot of 18 samples of broomcorn millet at seedling stage. (**B**) PCA plot of 16 samples of broomcorn millet at filling stage excluding two outliers (WNM8F-2 and ZLN5F-3). Samples of wild types, landraces, and improved cultivars are indicated in red, green, and blue symbols, respectively. WS, LS, and IS indicate wild types, landraces, and improved cultivars at seedling stage, respectively; WF, LF, and IF present wild types, landraces, and improved cultivars at filling stage, respectively.

To ensure the reproducibility of the biological replicates and to detect abnormal samples, a principal component analysis (PCA) was performed using the fragments per kilobase of transcript per million fragments mapped (FPKM) values of genes identified from the 18 samples at seedling stage and 18 samples at filling stage, respectively. The results showed that after the removal of two outliers, i.e., a wild type sample (WNM8F-2) and a landrace sample (LLN5F-3) at filling stage, the groups of wild types (WS and WF) and landraces (LS and LF) were well established and separated from each other, while the groups of improved cultivars (IS and IF) were largely mixed with those of landraces (LS and LF) (Figure 1A,B). These two samples of outliers were excluded from subsequent analyses.

### 2.2. Gene Expression and Co-Expression Network

**Figure 2:**
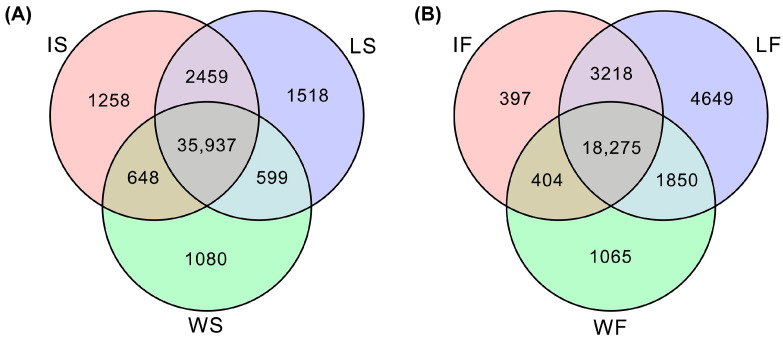
Venn diagram of genes expressed in wild types, landraces, and improved cultivars of broomcorn millets at seedling stage (**A**) and filling stage (**B**). WS, LS, and IS indicate wild types, landraces, and improved cultivars at seedling stage, respectively; WF, LF, and IF present wild types, landraces, and improved cultivars at filling stage, respectively.

A total of 43,499 and 29,858 genes (FPKM value ≥ 1) were expressed in at least one of the 18 samples at seedling stage and 16 samples at filling stage, respectively (Figure 2). Specifically, there were a total of 38,264, 40,513, and 40,302 genes expressed in at least one sample of WS, LS, and IS, respectively, and there were 21,594, 27,992 and 22,294 genes expressed in at least one sample of the WF, LF, and IF, respectively. In addition, a total of 1080, 1518, and 1258 genes were exclusively expressed in samples of WS, LS, and IS, respectively, and there were a total of 1065, 4649, and 397 genes uniquely detected in samples of WF, LF, and IF, respectively. Moreover, the number of co-expressed genes in samples of both seedling and filling stages reached 35,937 and 18,275, respectively, suggesting a higher similarity of gene expression among wild types, landraces, and improved cultivars of broomcorn millet at seedling stage than that of filling stage.

**Figure 3:**
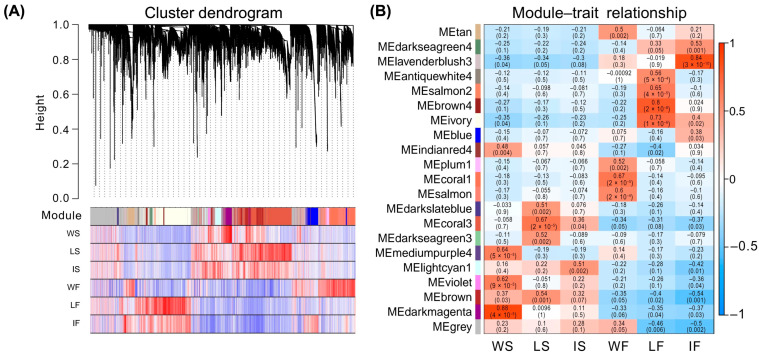
Detection of co-expression network in experimental groups of wild types, landraces, and improved cultivars of broomcorn millets at seedling and filling stages. (**A**) Hierarchical clustering tree of genes with assigned modules indicated. The main tree branches constitute 21 modules labeled by different colors, and the genes are represented by the leaves in the branches. The gray module is reserved for genes not assigned to any of the other 20 modules. WS, LS, and IS indicate wild types, landraces, and improved cultivars at seedling stage, respectively, and WF, LF, and IF present wild types, landraces, and improved cultivars at filling stage, respectively. (**B**) Associations between 21 modules (21 rows) and 6 traits (6 columns), i.e., WS, LS, IS, WF, LF, and IF. Each cell at the row–column intersection contains the correlation coefficient between the corresponding module and trait as well as the corresponding *p* values given in parentheses and are color-coded based on the color legend.

A WGCNA was then performed to identify a total of 21 modules (i.e., clusters of genes that exhibit highly correlated expression patterns across different samples) containing 23,299 genes associated with six broomcorn millet groups, i.e., WS, LS, IS, WF, LF, and IF (Figure 3A). The results showed that there were three (mediumpurple4, violet, and darkmagenta), four (darkslateblue, coral3, darkseagreen3, and brown), one (lightcyan1), four (tan, plum1, coral1, and salmon), four (antiquewhite4, salmon2, brown4, and ivory), and two (darkseagreen4 and lavenderblush3) co-expression modules with a module significance (MS) score > 0.5 and *p* < 0.01, which were significantly correlated with experimental groups WS, LS, IS, WF, LF, and IF, respectively (Figure 3B). It was noted that more modules showed a significant association with groups of wild types (WS and WF) and landraces (LS and LF) than those related to groups of improved cultivars (IS and IF) at both seedling and filling stages. Additionally, it was observed that the modules related to seedling stage (WS, LS, and IS) evidently differed from those related to filling stage (WF, LF, and IF) (Figure 3B).

The hub genes (i.e., the key genes within a co-expression network that play a central role in regulating biological processes) with gene significance (GS) score > 0.2 and MS score > 0.7 revealed in these 18 significantly correlated modules were chosen for subsequent analysis. A total of 956, 4570, 846, 3092, 3472, and 467 hub genes were detected as highly accumulated in groups of WS, LS, IS, WF, LF, and IF, respectively. As a result, a total of 5526 and 6564 hub genes specifically related to WS/LS and WF/LF were considered possibly related to the domestication of broomcorn millet, while 5416 and 3939 hub genes significantly correlated with LS/IS and LF/IF were identified as candidate genes associated with the improvement of broomcorn millet.

### 2.3. Functional Annotation and Enrichment Analysis of DEGs

**Figure 4:**
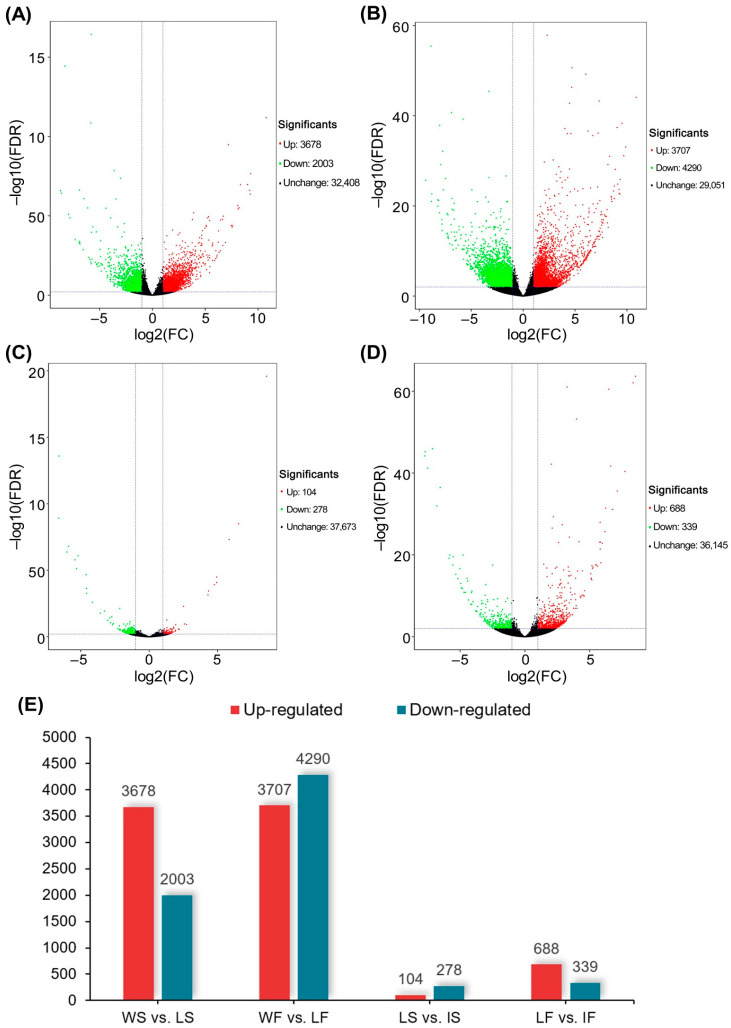
Differential gene expression analysis between wild types and landraces as well as between landraces and improved cultivars of broomcorn millet at seedling and filling stages, showing volcano plots of gene expression variation between WS and LS (**A**), WF and LF (**B**), LS and IS (**C**), and LF and IF (**D**). Each dot represents a gene, with genes up-regulated and down-regulated indicated in red and green, respectively; black dots represent the genes showing no significant difference. (**E**) Histogram showing the number of differentially expressed genes (DEGs) up-regulated and down-regulated in WS vs. LS, WF vs. LF, LS vs. IS, and LF vs. IF. Two vertical dashed lines represent |fold change| = 2; the horizontal dashed line represents significant *p* value of 0.05. WS, LS, and IS indicate wild types, landraces, and improved cultivars at seedling stage, respectively; WF, LF, and IF present wild types, landraces, and improved cultivars at filling stage, respectively.

There were a total of 5681 (3678 up-regulated and 2003 down-regulated), 7997 (3007 up-regulated and 4290 down-regulated), 382 (104 up-regulated and 278 down-regulated), and 1027 (688 up-regulated and 339 down-regulated) DEGs detected, based on a false discovery rate (FDR) < 0.01 and |fold change (FC)| ≥ 2, in the comparative analyses of WS vs. LS, WF vs. LF, LS vs. IS, and LF vs. IF, respectively (Figure 4A–D). It was noted that the number of DEGs in wild types vs. landraces (WS vs. LS and WF vs. LF) was obviously much higher than that in landraces vs. improved cultivars (LS vs. IS and LF vs. IF) at both seedling and filling stages, while the total number of DEGs detected in samples at filling stage (WF vs. LF and LF vs. IF) was slightly higher than that of the samples at seedling stage (WS vs. LS and LS vs. IS) (Figure 4E).

**Figure 5:**
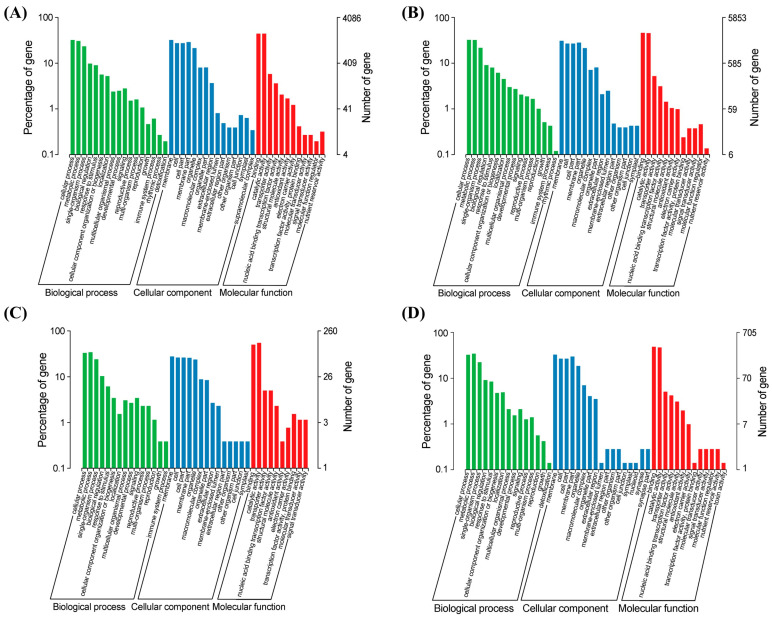
Gene Ontology (GO) annotation of differentially expressed genes (DEGs) detected in WS vs. LS (**A**), WF vs. LF (**B**), LS vs. IS (**C**), and LF vs. IF (**D**) of broomcorn millet. *X*-axis represents the GO terms of three categories, i.e., biological process, cellular component, and molecular function. *Y*-axis represent the number of DEGs annotated to the GO term (right) and percentage of number of DEGs annotated in all DEGs (left). WS, LS, and IS indicate wild types, landraces, and improved cultivars at seedling stage, respectively; WF, LF, and IF present wild types, landraces, and improved cultivars at filling stage, respectively.

A Gene Ontology (GO) annotation analysis was performed to reveal the functions of DEGs detected in broomcorn millets during the domestication and improvement processes. A total of 4086, 5853, 260, and 705 DEGs based on WS vs. LS, WF vs. LF, LS vs. IS, and LF vs. IF, respectively, were annotated to three categories of GO terms, i.e., biological processes, cellular components, and molecular function (Figure 5). The results revealed largely the same variation patterns of gene expression during the domestication and improvement of broomcorn millet, whereas the total number of DEGs annotated to each GO term was much higher during domestication than that of improvement at both seedling and filling stages. Specifically, for all four sets of DEGs based on WS vs. LS, WF vs. LF, LS vs. IS, and LF vs. IF, the top three GO terms with the highest number of DEGs annotated in the category of biological processes included cellular process (1311 genes, 32%; 1896 genes, 32%; 86 genes, 33%; 230 genes, 33%), metabolic process (1256, 31%; 1883, 32%; 89, 34%; 243, 34%), and single-organism process (959, 23%; 1269, 22%; 63, 24%; 160, 23%). The top five GO terms with the highest number of DEGs annotated in the category of cellular component were membrane (1315, 32%; 1802, 31%; 72, 28%; 232, 33%), cell (1125, 28%; 1577, 27%; 68, 26%; 191, 27%), cell part (1125, 28%; 1577, 27%; 68, 26%; 191, 27%), membrane part (1188, 29%; 1655, 28%; 67, 26%; 210, 30%), and organelle (875, 21%; 1252, 21%; 62, 24%; 132, 19%). In the category of molecular function, the top two GO terms annotated with the highest number of DEGs included binding (1806, 44%; 2703, 46%; 130, 50%; 344, 49%) and catalytic activity (1802, 44%; 2656, 45%; 142, 55%; 332, 47%).

**Figure 6:**
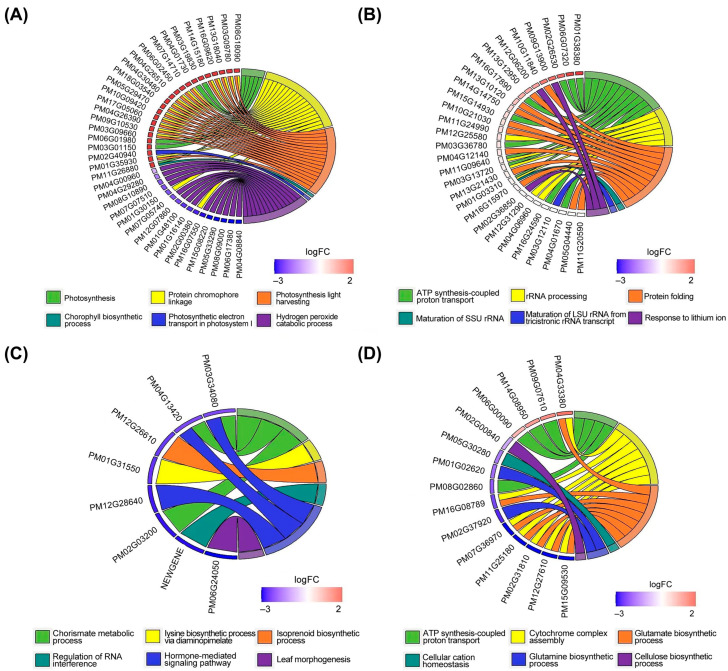
Chord plot of GO enrichment analysis based on differentially expressed genes (DEGs) in WS vs. LS (**A**), WF vs. LF (**B**), LS vs. IS (**C**), and LF vs. IF (**D**) of broomcorn millet. The enriched DEGs are linked via ribbons to their assigned GO terms. The enriched DEGs are arranged according to log (Fold Change), which is displayed as a blue-to-red coding scheme next to the selected genes. The up-regulated and down-regulated genes are indicated in red and blue, respectively, and the color intensity represents the relative value of fold change. WS, LS, and IS indicate wild types, landraces, and improved cultivars at seedling stage, respectively; WF, LF, and IF present wild types, landraces, and improved cultivars at filling stage, respectively.

Then, a GO enrichment analysis based on DEGs was further performed to reveal the association between the enriched GO terms and the corresponding inclusion of DEGs (Figure 6). The results of a GO chord plot showed that a total of 23 up-regulated and 16 down-regulated DEGs of WS vs. LS were enriched in the top six GO terms (i.e., photosynthesis, protein chromophore linkage, photosynthesis light harvesting, chlorophyll biosynthetic process, photosynthetic electron transport in Photosystem I, and hydrogen peroxide catabolic process) (Figure 6A). Similarly, a total of 29 up-regulated DEGs of WF vs. LF were involved in the top six GO terms (including ATP synthesis-coupled proton transport, rRNA processing, protein folding, maturation of SSU rRNA, maturation of LSU rRNA from tricistronic rRNA transcript, and response to lithium ion). There were a total of eight down-regulated DEGs of LS vs. IS enriched in the top six GO terms (i.e., chorismate metabolic process, lysine biosynthetic process via diaminopimelate, isoprenoid biosynthetic process, regulation of RNA interference, hormone-mediated signaling pathway, and leaf morphogenesis). For DEGs of LF vs. IF, there were four up-regulated and eleven down-regulated DEGs enriched in the top six GO terms (ATP synthesis-coupled proton transport, cytochrome complex assembly, glutamate biosynthetic process, cellular cation homeostasis, glutamine biosynthetic process, and cellulose biosynthetic process). These results showed that there were much more enriched DEGs related to domestication (WS vs. LS and WF vs. LF) than those involved in improvement (LS vs. IS and LF vs. IF) of broomcorn millet. Furthermore, most domestication-related DEGs were up-regulated, while most improvement-related DEGs were down-regulated. For the up-regulated DEGs related to domestication, DEGs of WS vs. LS were mainly enriched in GO terms related to photosynthesis, while the DEGs of WF vs. LF were mainly enriched in GO terms related to rRNA synthesis, maturation, and transcription. It was also noted that the GO term ‘ATP synthesis-coupled proton transport’ was enriched by DEGs of both WF vs. LF and LF vs. IF, but not enriched by DEGs of WS vs. LS or LS vs. IS, indicating that this GO term was probably related to the filling stage of broomcorn millets during either domestication or improvement processes.

**Figure 7:**
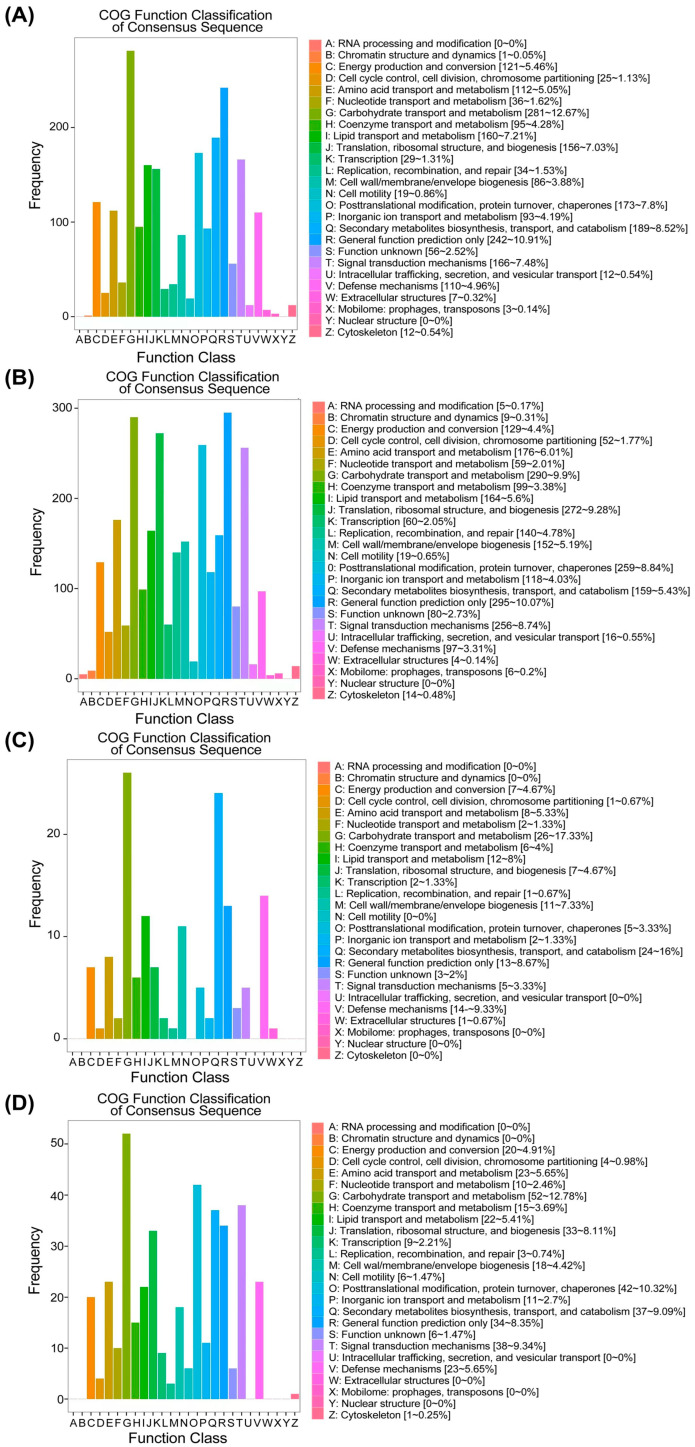
COG classification of differentially expressed genes (DEGs) detected in WS vs. LS (**A**), WF vs. LF (**B**), LS vs. IS (**C**), and LF vs. IF (**D**) of broomcorn millet. WS, LS, and IS indicate wild types, landraces, and improved cultivars at seedling stage, respectively; WF, LF, and IF present wild types, landraces, and improved cultivars at filling stage, respectively.

The Clusters of Orthologous Groups of proteins (COG) database was then used to identify the functional categories of DEGs detected in broomcorn millet. A total of 2218, 2930, 150, and 407 DEGs of WS vs. LS, WF vs. LF, LS vs. IS, and LF vs. IF were annotated up to 25 COG terms with an E-value ≤ 1 × 10^−5^ (Figure 7). The results largely revealed similar biological functions of DEGs related to the domestication and improvement processes of broomcorn millets. However, the number of DEGs related to domestication was higher than that of improvement at both seedling and filling stages. Specifically, for three out of the four sets of DEGs (except for WF vs. LF), the COG term ‘carbohydrate transport and metabolism prediction’ was annotated with the largest number of DEGs (281 genes, 13%; 290 genes, 10%; 26 genes, 17%; 52 genes, 13%), while the number of genes annotated to the COG term ‘carbohydrate transport and metabolism prediction’ was slightly less than that enriched in the COG term ‘general function prediction only’ in WF vs. LF. Additionally, several other COG terms annotated by high proportions of DEGs in all four sets of DEGs included ‘energy production and conversion’ (121, 5%; 129, 4%; 7, 5%; 20, 5%), ‘amino acid transport and metabolism’ (112, 5%; 176, 6%; 8, 5%; 23, 6%), ‘coenzyme transport and metabolism’ (95, 4%; 99, 3%; 6, 4%; 15, 4%), ‘lipid transport and metabolism’ (160, 7%; 164, 6%; 12, 8%; 22, 5%), ‘translation, ribosomal structure and biogenesis’ (156, 7%; 272, 9%; 7, 5%; 33, 8%), ‘cell wall/membrane/envelope biogenesis’ (86, 4%; 152, 5%; 11, 7%%; 18, 4%), ‘posttranslational modification, protein turnover, chaperones’ (173, 8%; 259, 9%; 5, 3%; 37, 9%), ‘secondary metabolites biosynthesis, transport and catabolism’ (189, 8%; 159, 5%; 24, 16%; 37, 9%), ‘general function prediction only’ (242, 11%; 295, 10%; 13, 9%; 34, 8%), ‘signal transduction mechanisms’ (166, 7%; 256, 9%; 5, 3%; 38, 9%), and ‘defense mechanisms’ (110, 5%; 97, 3%; 14, 9%; 23, 6%).

**Figure 8:**
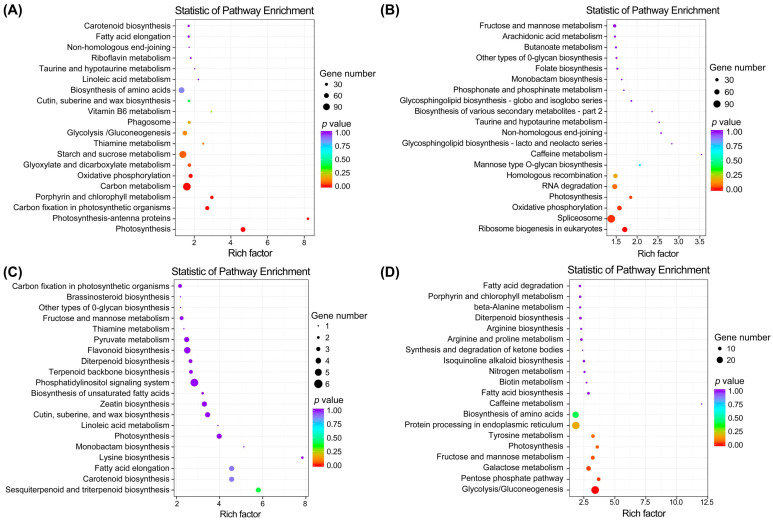
Kyoto Encyclopedia of Genes and Genomes (KEGG) metabolic pathway enrichment analysis of differentially expressed genes (DEGs) in WS vs. LS (**A**), WF vs. LF (**B**), LS vs. IS (**C**), and LF vs. IF (**D**) of broomcorn millet. The top 20 most enriched pathways are listed on *Y*-axis; the *X*-axis presents the enrichment factor based on the ratio of the proportion of the DEGs annotated to a pathway to the proportion of all genes annotated to the pathway. The color intensity of the dots stands for q value (adjusted *p* value), and the size of the dots represents the number of DEGs enriched in the pathway. WS, LS, and IS indicate wild types, landraces, and improved cultivars at seedling stage, respectively; WF, LF, and IF present wild types, landraces, and improved cultivars at filling stage, respectively.

Furthermore, a Kyoto Encyclopedia of Genes and Genomes (KEGG) pathway enrichment analysis of DEGs was performed to identify the significantly over-expressed metabolic pathways in broomcorn millet. The results showed that among the significantly enriched pathways (*p* value ≤ 0.05), ‘photosynthesis’ and ‘oxidative phosphorylation’ were enriched by DEGs of both WS vs. LS and WF vs. LF (Figure 8A,B). In addition, the DEGs of WS vs. LS were also enriched in several other pathways, e.g., ‘photosynthesis-antenna proteins’, ‘carbon fixation in photosynthetic organisms’, ‘porphyrin and chlorophyll metabolism’, ‘carbon metabolism’, and ‘glyoxylate and dicarboxylate metabolism’, while the DEGs of WF vs. LF were enriched in other two pathways, i.e., ‘ribosome biogenesis in eukaryotes’ and ‘spliceosome’. Additionally, no pathways were significantly enriched by DEGs of LS vs. IS, while the DEGs of LF vs. IF were mainly enriched in pathways of ‘glycolysis/gluconeogenesis’, ‘pentose phosphate pathway’, ‘galactose metabolism’, and ‘fructose and mannose metabolism’. These results revealed varied metabolic pathways specifically related to the domestication and improvement processes of broomcorn millet.

### 2.4. Screening of Candidate Genes Related to Domestication and Improvement of Broomcorn Millet

The results of the WGCNA and differential gene expression analysis identified the candidate genes related to the domestication and improvement processes of broomcorn millet, i.e., the intersection of the hub genes based on the WGCNA and the DEGs obtained through the differential gene expression analysis, respectively. In total, 2155 and 3033 candidate genes involved in domestication and a total of 84 and 180 candidate genes related to improvement were obtained at seedling and filling stages of broomcorn millet, respectively. The annotation analysis of these genes based on various databases was performed to identify the functions of these DEGs (Table S2). These results revealed much more candidate genes related to domestication than those involved in the improvement of broomcorn millet, while the number of candidate DEGs at seedling stage was slightly less than that at filling stage. Moreover, the 2486 domestication-related genes identified through a selective sweep analysis (i.e., a method used in population genetics to identify regions of the genome that have undergone positive selection) in our previous SLAF-Seq analysis of broomcorn millet were used to further screen more reliable candidate genes related to the domestication of broomcorn millet [53]. The results showed that a total of 14 and 29 candidate genes related to domestication at seedling and filling stages (Table S3), respectively, were identified by both comparative transcriptome analysis in this study and our previous selective sweep analysis based on the SLAF-Seq study. Specifically, the 14 candidate genes at seedling stage were annotated as the *CSI1*, *ILI4*, *NIP2-2*, *PER2*, *At5g01750*, *PLATZ* transcription factor-related gene, *YUC2*, *PLDZETA1*, *TSP9*, *P2*, *GAPB*, and *SGO1*, and the 29 candidate genes at filling stage were annotated as *PER51*, *TIM23-2*, sugar-transporter-related gene, *PYL10*, *GUX2*, the probable lipid transfer-related gene, *VQ9*, *ASG2*, *MIPS*, *ALY3*, *MYBS1*, *Os03g0216600*, *At1g32860*, *RLT3*, *HSP26.7*, *TIF3C1*, *At5g01750*, *ANT1*, *Os04g0620700*, *PPOX1*, *Urb2*, *PILS7*, *CHR24*, *Os04g0620700*, *HSP26.7*, and *At1g09820*, as well as a few uncharacterized or hypothetical genes.

### 2.5. Validation of Gene Expression Based on RNA-Seq Using qRT-PCR

**Figure 9:**
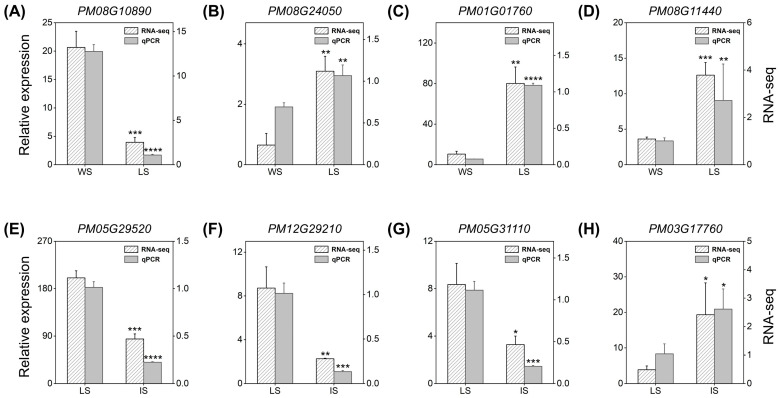
Quantitative real-time PCR (qRT-PCR) validation of expression levels of eight genes based on WS vs. LS (**A**–**D**) and LS vs. IS (**E**–**H**). *Y*-axis shows the relative gene expression levels based on qRT-PCR (**left**) and RNA-Seq (**right**). The expression levels of genes based on qRT-PCR are normalized by *18S rRNA* and determined using the 2^–ΔΔCt^ method. The significant difference in the gene expression levels between WS vs. LS and between LS vs. IS was determined using Student’s *t*-test based on *p* < 0.05 (*), *p* < 0.01 (**), *p* < 0.001 (***), and *p* < 0.0001 (****), respectively. WS, LS, and IS indicate wild types, landraces, and improved cultivars at seedling stage, respectively.

A total of eight genes (four DEGs related to domestication and four related to improvement) were selected for further qRT-PCR validation of the gene expression patterns based on an Illumina RNA-Seq analysis (Figure 9). The results revealed the consistent expression patterns of these genes between qRT-PCR and RNA-Seq analyses, i.e., four genes (*PM08G24050*, *PM08G11440, PM01G01760,* and *PM03G17760*) were significantly up-regulated and the other four genes (*PM08G10890, PM05G29520, PM12G29210,* and *PM05G31110*) were significantly down-regulated.

## 3. Discussion

In this study, the comparative transcriptome analysis was performed between wild types and landraces and between landraces and improved cultivars of broomcorn millet at seedling and filling stages, respectively. To our knowledge, this is the first report on the systematically comparative transcriptome investigation of different evolutionary types of broomcorn millet at different developmental stages. The DEGs identified in the comparative analysis of wild types vs. landraces and landraces vs. improved cultivars provided us abundantly essential genetic materials for future studies of domestication and improvement of broomcorn millet. Furthermore, the use of plant samples at both seedling and filling stages provided novel insights into the gene expression variations of broomcorn millets among different developmental stages.

The results of the PCA based on gene FPKM values showed that the landraces were well separated from wild types, but largely mixed with improved cultivars (Figure 1) of broomcorn millet. The differential gene expression analysis also revealed much more DEGs related to domestication than those associated with improvement (Figure 4E), and the intersection analysis of WGCNA and the differential gene expression analysis identified a total of 5188 and 264 candidate genes related to the domestication and improvement of broomcorn millet, respectively. Together, these results suggested higher selection pressure and gene expression variations during the domestication of broomcorn millet than those during improvement, which was probably due to the long domestication process and the relatively recent, short improvement process of broomcorn millets that started in the 1940s in China [41]. Given the abundant resources of wild types and landraces of broomcorn millet, as well as the insufficient improvement and strong market demand of this crop, it is reasonably expected that significant efforts are still required in the future to improve broomcorn millet with enhanced yield and quality.

In our study, the initial set of 5188 domestication-related genes were further screened and narrowed down to a total of 43 more reliable candidate genes by comparing with the 2486 domestication-related genes identified through our previous selective sweep analysis of broomcorn millets based on SLAF-Seq [53]. In particular, the majority of the 43 domestication-related genes and 264 improvement-related genes were mainly annotated to metabolite biosynthesis, transport, and catabolism, stress resistance to salt, disease, heat, as well as plant hormone signal transduction (Table S3), strongly suggesting that the genes related to metabolites, stress resistance, and plant hormones were widely selected. However, it was noted that some DEGs of the same gene families were exclusively detected in either domestication or improvement processes. For example, *PM08G24050, PM07G06370,* and *PM06G29860,* which were annotated as *YUC2* and *YUC3* of *Arabidopsis thaliana* and *YUC1* of *Oryza sativa* subsp. *japonica* [64,65], respectively, all in the *YUCCA* gene family and encoding indole-3-pyruvate monooxygenase YUCCA involved in the promotion of auxin synthesis, were only detected at seedling stage during domestication. These results suggested that the *YUC* genes may have been selected during domestication and these genes are mainly highly expressed during the seedling stage of broomcorn millet. The up-regulation of *PM08G24050* and *PM07G06370* was consistent with the previous study, showing that the cultivated broomcorn millets were taller than the wild types of broomcorn millet during seedling stage [41]. However, the gene *PM06G29860*, which was annotated to *YUC1* of *O. sativa* subsp. *japonica*, was down-regulated during the seedling stage of domestication. Previous studies showed that rice plants with an overexpression of *OsYUC1* exhibited the characteristics of inhibited leaf growth and root elongation but promoted the formation of crown root [65]. Therefore, the expression level of this gene was probably reduced during domestication in order to adapt to the cultivation of broomcorn millet. Additionally, a group of six genes, i.e., *PM07G30560, PM07G34640, PM15G17180, PM12G22500, PM01G20870,* and *PM15G19420,* annotated to *SCL1, SCL3, SCL6, SCL6, SCL6,* and *SCL8,* respectively, of the *GRAS* gene family in *A. thaliana* [48], were only detected downregulated during the filling stage of domestication. Studies showed that *SCL6* of *A. thaliana* is involved in the negative regulation of shoot branching [66]. Therefore, the downregulation of these genes was probably related to the regulation of plant type and panicle type during domestication. Further studies are necessary to verify these findings revealed in our study. Furthermore, a few candidate genes related to waterlogging tolerance were only upregulated at filling stage during the improvement process. For instance, both genes *PM18G05050* and *PM17G07290* were annotated as *ADH1*, which was the waterlogging responsive alcohol dehydrogenase gene in *Actinidia deliciosa* [67]; two genes (*PM02G29240* and *PM05G34990*) were annotated as *PDC1*, which was involved in waterlogging stress in *Arabidopsis thaliana* [68]; and two genes (*PM01G11930* and *PM02G29240*) were annotated as *PDC2*, which was a waterlogging and heat stress tolerance gene in *Actinidia deliciosa* [69]. The upregulated expression of these waterlogging tolerance genes during the improvement process was probably due to the requirements for artificial irrigation during the filling stage of cultivated broomcorn millets in order to achieve high yield. However, this crop is not tolerant to waterlogging, and improper irrigation could lead to waterlogging damage. Therefore, it was likely that these genes involved in waterlogging resistance were selected during the improvement process of broomcorn millet. These results indicated that both sets of same/similar or different genes were selected during the domestication and improvement stages of broomcorn millets, achieving the similar and different goals of selection during the domestication and improvement processes of broomcorn millet.

The morphological differences between weedy and cultivated broomcorn millet are mainly reflected in pericarp and seed color, plant height, panicle type, tiller number, and seed size [41,54,55]. Therefore, a comparative transcriptome analysis of wild type and landrace broomcorn millet was performed to identify genes related to these domestication traits. For example, *newGene_970*, annotated as *F3H,* was confirmed to be associated with a thicker grain coat in wheat [70]. Three genes (*PM08G24050, PM07G06370,* and *PM06G29860*) annotated as *YUC2* and *YUC3* in *Arabidopsis* and *YUC1* in rice were involved in plant height [64,65], while other three genes (*PM15G17180*, *PM12G22500*, and *PM01G20870*) annotated to *SCL6*, which was involved in the negative regulation of shoot branching in *Arabidopsis* [66], were probably associated with the panicle type of broomcorn millet. The identification of these genes significantly enriches the gene pool of molecular breeding of broomcorn millet. Furthermore, molecular and genetic analyses have confirmed the existence of strong domestication bottleneck from weedy to cultivated broomcorn millet [14,53]. Therefore, the comparative transcriptome analysis of wild type and landrace broomcorn millet is highly significant for the conservation and utilization of wild germplasm resources of this crop. Moreover, the differences between landraces and improved cultivars of broomcorn millet are mainly manifested in agricultural production, taste, nutrition, and stress resistance [41,56]. These differential traits are the focus of attention in the improvement of broomcorn millet, and a comparative transcriptome analysis of landraces and improved cultivars could identify genes associated with these traits. For example, two genes (*PM01G17520* and *PM02G23870*) were annotated as the *SUS1* gene (encoding sucrose synthase), which was involved in the synthesis of starch, callose, and cellulose, as well as in seed weight and phenotype in *Zea mays* [71]. The exploration of these genes provides powerful tools for future molecular breeding efforts in broomcorn millet. Further investigations are necessary to verify the findings revealed in the current study, i.e., a functional confirmation of the genes.

Our results revealed much more co-expressed genes in samples at seedling stage (35,937) than those at filling stage (18,275) (Figure 2), while the number of DEGs at seedling stage was slightly less than that at filling stage (Figure 4E). The intersection results of the WGCNA and differential gene expression analysis identified a total of 2199 and 3213 candidate genes related to both the domestication and improvement processes of broomcorn millet at seedling and filling stages, respectively. Additionally, a variety of co-expression network modules was detected between seedling and filling stages of broomcorn millet (Figure 3). The seedling stage of broomcorn millet spans from emergence to jointing, during which the primary focus was on root establishment and leaf growth. Because this stage is crucial for the growth and development of broomcorn millet, it is also a key period for drought resistance studies [72]. Therefore, focusing on genes during the seedling stage in this study could help identify genes related to the growth, development, and drought resistance of broomcorn millet. Furthermore, these genes are closely linked to the cultivation and yield of broomcorn millet, making them valuable for research. In our study, some candidate genes involved in plant growth and development as well as drought resistance were upregulated at seedling stage. For example, gene *PM01G01760* was annotated as *GAPB*, encoding glyceraldehyde-3-phosphate dehydrogenase B, an enzyme involved in the Calvin cycle of photosynthesis; *PM08G24050* and *PM07G06370* were annotated as *YUC2* and *YUC3*, respectively, which are important for the growth and development of broomcorn millet [64,65], while *PM16G06610* was annotated as *ACS*, encoding Acetyl-coenzyme A synthetase, which was related to drought resistance in willow [73]. The filling stage of broomcorn millet encompasses the period from heading to grain maturity. During this stage, the grains undergo rapid starch accumulation. It is a critical period that determines both the yield and quality of broomcorn millet [74]. Therefore, numerous genes related to domestication and improvement during the filling stage are closely linked to the yield and quality of broomcorn millet, making them important candidates for future molecular breeding. In our study, it was observed that the expression of some candidate genes related to seed yield and quality was upregulated at the filling stage. For instance, the gene *PM03G24050* was annotated as *ANT1*, encoding an AINTEGUMENTA transcription factor involved in the promotion of fruit enlargement [75,76]; both genes *PM01G17520* and *PM02G23870*, annotated as *SUS1* gene, are related to quality (starch, callose, and cellulose synthesis) and yield (seed weight and seed phenotype) of this crop [71]. Together, these results suggested that the selection mainly placed pressure on domestication- and improvement-related genes involved in both vegetative and reproductive growth at the seedling and filling stages of broomcorn millet, respectively. Moreover, higher selection pressure was placed on reproduction-related traits than on vegetative traits, i.e., both evolutionary and agricultural selections have placed greater emphasis on enhancing reproductive traits over vegetative traits, simply due to their direct influence on both quantity and quality of broomcorn millet seeds; these are crucial features for crop propagation and yield improvement. Future research is required to validate the functions of these genes during the reproductive and vegetative development of broomcorn millet.

It was noted that genes of broomcorn millet related to stress resistance were either upregulated or downregulated, implying that some stress resistance genes probably lost their function or reduced their expression level during domestication or improvement. For example, the gene *PM07G11550*, annotated as drought and cold stress tolerance gene *PYL10* in *Oryza sativa* subsp. *indica* [77], was downregulated at filling stage during the domestication of broomcorn millet. These results are consistent with the common agronomic observations, demonstrating that certain wild types of broomcorn millet exhibit higher stress resistance capability than cultivars. Similarly, previous studies showed that the wild genotypes frequently harbor more resistance genes than the cultivars in various plants, such as barley [78,79], rice [80], pear [81], watermelon [82], cucumber [83], grapevine [84], and cardamom [85]. These variations were probably due to the trade-offs between stress resistance and productivity during crop domestication. Previous studies showed that high levels of genetic immunity to disease were often observed accompanied by a reduction in the yield of crops. For example, the expression of streak mosaic virus R gene *Wsm1* from wheat was accompanied by a yield reduction of about 21% [86], the stem rust R gene *Sr26* from *Agropyron elongatum* led to a nearly 9% decrease in production [87], the barley *mlo R* gene showed a yield loss of 4.2% [88], and the *RPS5+* allele in *Arabidopsis thaliana* caused a fitness cost of 5–10.2% [89]. The crosstalk between plant development and disease resistance may be caused by genes that act as plasma membrane residents, a MAPK cassette, nuclear envelope channels components, and pleiotropic regulators [90]. Therefore, recent investigations focused on optimizing growth strategies to resolve the trade-offs between disease resistance and the high yield of crops [91]. To date, these strategies are not reported in broomcorn millet. The downregulated expression of stress resistance genes in landraces compared with wild types of broomcorn millets implies the trade-offs between stress resistance and a high yield in this crop, suggesting that in order to achieve high yields, it is necessary to compromise certain stress resistance traits and reduce the costs associated with plant growth and development during domestication. Therefore, it is crucial to conserve both the wild and landrace resources of broomcorn millet and to further explore the functions of their stress resistance genes. These efforts would definitely provide valuable genetic materials for future molecular breeding of broomcorn millet of varieties with an optimal balance between stress resistance and plant growth and development, ultimately enhancing both reproductive and vegetative traits in broomcorn millet.

The limitations of this study were noted. First, this study mainly investigated the variations in gene expression during the domestication and improvement processes of broomcorn millet based on transcriptome sequencing. It is expected that, in combination with the analysis of gene sequence variation, more comprehensive and reliable candidate genes related to the domestication and improvement of broomcorn millet could be identified. Second, this study only focused on the domestication process of broomcorn millet to comprehensively screen the results of expression variation and sequence variation to identify the relatively reliable candidate genes based on the results of our previous selection sweep analysis of SLAF-Seq. However, the SLAF-Seq analysis was performed using a simplified genome, i.e., the entire genome of broomcorn millet was not covered, ultimately unavoidably missing some candidate genes during the comparative analysis. Therefore, the selective sweep analysis based on whole-genome sequencing should provide more valuable candidate genes. Third, it is still necessary to verify the molecular functions of the genes identified in the current study during the reproductive and vegetative growth of broomcorn millet.

## 4. Materials and Methods

### 4.1. Plant Materials

A total of 36 samples of broomcorn millet plants were selected for transcriptome profiling (Table S1), including two accessions of wild type (WNX24 and WNM8), two accessions of landraces (LLN5 and LJL8), and two accessions of improved cultivars (ISX63 and IGS169), with three individual plants for each accession at both seedling and filling stages, respectively. Based on the evolutionary types and development stages, these samples were divided into six experimental groups, i.e., wild types at seedling (WS) and filling (WF) stages, landraces at seedling (LS) and filling (LF) stages, and improved cultivars at seedling (IS) and filling (IF) stages. The seeds of all these accessions of broomcorn millet used in this study were provided by the Institute of Crop Science, Chinese Academy of Agricultural Sciences (ICSCAAS), Beijing, China, and sown and grown at an experimental field under natural conditions in May 2020 (Jilin Agricultural University, Changchun, China). The wild type accessions were identified based on relevant morphological characteristics, i.e., pericarp color, plant height, panicle type, tiller number, seed color, and seed size [41,54,55]. The samples at seedling stage, comprising shoots and roots of each accession, were collected when the second leaf first emerged, while the samples at filling stage, consisting of roots, stems, leaves, and seeds of each accession, were harvested 15 d after flowering. All these plant organs with equal weights (approximately 2 g) were collected, quickly frozen in liquid nitrogen, and then stored at −80 °C for RNA extraction.

### 4.2. RNA Extraction, cDNA Library Construction, and Sequencing

Total RNA was extracted from the whole plants at seedling stage and from a mixture of roots, stems, leaves, and seeds of equal weight at filling stage using an RNAprep Pure Plant Kit (Tiangen, Beijing, China). RNA purity and concentration were examined by the NanoDrop 2000 (Thermo Fisher Scientific, Wilmington, DE, USA). RNA integrity was measured using the RNA Nano 6000 Assay Kit of the Agilent Bioanalyzer 2100 system (Agilent Technologies, Santa Clara, CA, USA). RNA samples with high quality were selected to construct the cDNA libraries using the NEBNext UltraTM RNA Library Prep Kit for Illumina (NEB; Ipswich, MA, USA), following the manufacturer’s instructions. The library quality was assessed using the Agilent Bioanalyzer 2100 system (Agilent Technologies, Santa Clara, CA, USA). After cluster generation, the library preparations were sequenced on the Illumina HiSeq platform (Illumina, San Diego, CA, USA) by the Biomarker Technologies Corporation (Beijing, China).

### 4.3. Read Mapping, Transcript Assembly, and SNP Calling

Clean data of RNA-Seq with high quality were obtained by removing reads containing adapters, reads containing N (i.e., uncertain nucleotide) more than 10%, and reads with more than 50% low-quality score nucleotides (Q ≤ 10) from raw data. These clean data were then mapped onto a broomcorn millet reference genome (https://www.ncbi.nlm.nih.gov/assembly/GCA_003046395.2/; accessed on 25 May 2023) using HISAT2 2.2.1 with parameters “--dta -p 6 --max-intronlen 5000000” [92,93]. Reads with either a perfect match or one mismatch were further analyzed and annotated based on the reference genome. Both Picard-tools v1.41 and SAMtools 0.1.18 were used to sort and remove duplicated reads and to merge the BAM alignment results of each sample. GATK 2.0 software was used to perform SNP calling using parameters “-dontUseSoftClippedBases -stand_call_conf 20.0 -stand_emit_conf 20.0” [94]. Raw vcf files were filtered using the GATK standard filter method with parameters “clusterWindowSize: 10; MQ0 >= 4 and (MQ0/(1.0*DP)) > 0.1; QUAL < 30.0 || QD < 5.0 || HRun > 5” to retain SNPs with distance > 5.

### 4.4. Gene Expression and Weighted Gene Co-Expression Network Analysis

The quantification of gene expression levels was estimated by fragments per kilobase of transcript per million fragments mapped (FPKM) method using StringTie 1.3.3b based on a maximum flow algorithm [95]. A principal component analysis (PCA) on gene expression was used to eliminate sample outliers. The WGCNA 1.14.0 was performed to assess the co-expression networks of genes responsible for the variations among the six traits [96], i.e., the six experimental groups of WS, LS, IS, WF, LF, and IF. The modules were obtained using the automatic network construction function blockwiseModules with the following settings: power = 26, TOMType = unsigned, minModuleSize = 30, and mergeCutHeight = 0.25. The genes with high pairwise correlation coefficients were clustered together as a module. Then, the gene significance (GS) and the module significance (MS) scores were calculated to identify the modules associated with relevant traits. The parameters “cor.geneTraitSignificance > 0.2” and “cor.geneModuleMembership > 0.7” were used to identify hub genes with high significance for relevant traits as well as high module membership in relevant modules. The hub genes specifically related to WS/LS and WF/LF were considered associated with the domestication of broomcorn millet, while the hub genes significantly correlated with LS/IS and LF/IF were defined as being related to the improvement of broomcorn millet.

### 4.5. Differential Gene Expression Analysis and Functional Annotation and Enrichment Analyses of Differentially Expressed Genes

DEGs related to the domestication and improvement processes were detected in the comparative analyses of WS vs. LS, WF vs. LF, LS vs. IS, and LF vs. IF, respectively, using DESeq2 1.44.0 based on a false discovery rate (FDR) < 0.05 and |Fold Change (FC) ≥ 2| [97]. Then, the DEGs were annotated using databases of non-redundant (Nr) protein sequences at the National Center for Biotechnology Information (NCBI; http://www.ncbi.nlm.nih.gov/blast/; accessed on 20 June 2023) [98], protein family (Pfam; http://pfam.xfam.org/; accessed on 10 July 2023) [99], Swiss-Prot protein sequence (http://www.uniprot.org/; accessed on 13 July 2023) [100], Clusters of Orthologous Groups of proteins (COG; http://www.ncbi.nlm.nih.gov/COG/; accessed on 20 July 2023) [101], Clusters of protein homology (KOG; http://www.ncbi.nlm.nih.gov/COG/; accessed on 5 August 2023) [102], evolutionary genealogy of genes: Non-supervised Orthologous Groups 5.0 (eggNOG 5.0; http://eggnog5.embl.de/; accessed on 8 August 2023) [103], Gene Ontology (GO; http://www.geneontology.org/; accessed on 16 August 2023) [104], and Kyoto Encyclopedia of Genes and Genomes (KEGG; http://www.genome.jp/kegg/; accessed on 25 August 2023) [105]. GO enrichment analyses of DEGs were implemented by the GOseq 1.56.0 R packages based on a Wallenius non-central hyper-geometric distribution to adjust the gene length bias [106]. GO chord plots were generated by GOplot 1.0.2 R packages based on ggplot2 3.4.4 [107]. A KEGG pathway enrichment analysis of DEGs was performed using the KO-Based Annotation System (KOBAS 2.0) [108].

Subsequently, the common genes shared between hub genes based on the WGCNA and DEGs related to domestication and improvement during seedling and filling stages of broomcorn millet, respectively, were defined as candidate domestication- and improvement-related genes, respectively. Additionally, these candidate domestication-related genes were further compared with the 2486 domestication-related genes identified through our previous selective sweep analysis based on the SLAF-Seq of broomcorn millet [53] to screen more reliable candidate genes.

### 4.6. Validation of Gene Expression with Quantitative Real-Time PCR

A total of 8 DEGs during the seedling stage of broomcorn millet were randomly selected for further validation using quantitative real-time PCR (qRT-PCR), with *18S rRNA* used as the internal reference gene. The gene-specific primers were designed using Primer 3 program (http://bioinfo.ut.ee/primer3-0.4.0/; accessed on 2 September 2023; Table 1). Each experiment was repeated with three biological replicates. Each RNA sample (1 μg) was reverse transcribed using SynScript^®^III RT SuperMix for qPCR (TSK314S) (Tsingke, Beijing, China) according to the manufacturer’s instructions. The qRT-PCR was performed using ArtiCanCEO SYBR qPCR Mix (TSE401) (Tsingke, Beijing, China) with an QuantStudio Stepone Plus Real-time PCR System (ABI, Foster City, CA, USA). The PCR amplification was performed as follows: denaturation at 95 °C for 5 min, followed by 40 cycles of denaturation at 95 °C for 15 s, annealing at 60 °C for 20 s, and extension at 72 °C for 20 s. To verify the amplification specificity, melting curve analysis was performed following amplification. The melting curve range was extended from 65 °C to 95 °C, with an increment of 0.1 °C/s. The relative gene expression levels were determined using the 2^–ΔΔCt^ method based on three biological replicates.

## 5. Conclusions

To sum up, the comparative transcriptome analysis was performed based on wild types, landraces, and improved cultivars of broomcorn millet at seedling and filling stages, respectively. The integration of gene co-expression network analysis and differential gene expression analysis, as well as our previous selective sweep analysis based on SLAF-Seq in broomcorn millet, provided us a group of candidate genes specifically related to domestication and improvement processes of broomcorn millet. The acquisition of these candidate genes provides valuable experimental evidence and genetic materials for the studies of both domestication and improvement of broomcorn millet, laying a solid foundation for the future cultivation and molecular breeding of broomcorn millet, as well as further exploration of the molecular mechanisms underlying the domestication and improvement processes of this crop.

## Figures and Tables

**Table 1:** Primers and their sequences used in qRT-PCR analysis. “F” and “R” represent the forward and reveres primers, respectively.

Gene	Primer Sequence (5′–3′)
*PM08G10890*	F: CGTCATCAACCACCGCCTAT R: GTGTGCGTGTTGAATTGGCT
*PM08G24050*	F: AGCAATGTTCCCTTCTGGCT R: GGCCATTCTCACCCTTCCAC
*PM01G01760*	F: GGTTTGCTGAGAGAGACGGA R: ACACACAGCAGTCCCAACAG
*PM08G11440*	F: CGGAAGGAGAAAGTCAGCCA
	R: GCAACTGCTCCATAGGGTCA
*PM05G29520*	F: ACGTCCCAGGTTCCTCAAAG R: CCGACTTCTGGTGGTAGCAG
*PM12G29210*	F: CGGCGAGGACAACATGGAGTA R: CCATCGTGTCGTGGATTCGAG
*PM05G31110*	F: CGCAGCCTAACGAGAACGAT R: GCATTTCAGCAGTGAGCGAG
*PM03G17760*	F: CACCGACTACGACGGCTACA
	R: AGACGACGCGCAGGTAGATG
*18S rRNA*	F: GCGAGTACGGTTCGGATTGA R: CTCATGCGCCAATGCTACAC

## Data Availability

The raw RNA-Seq data were deposited at the National Center for Biotechnology Information (NCBI; https://www.ncbi.nlm.nih.gov/bioproject/; accessed on 30 July 2024) database with an accession of PRJNA1141739.

## Supplementary Materials

The following supporting information can be downloaded at: https://www.mdpi.com/article/10.3390/ijms252011012/s1, Table S1: Statistics of RNA-seq data of wild accessions, landraces, and improved cultivars of *Panicum miliaceum* L. at both seedling and filling stages used in this study. WS, LS, and IS indicate wild types, landraces, and improved cultivars at seedling stage, respectively; WF, LF, and IF present wild types, landraces, and improved cultivars at filling stage, respectively; Table S2: Annotation analyses of differentially expressed genes (DEGs) based on various databases; Table S3: List of 43 domestication-related genes and 264 improvement-related genes identified in broomcorn millet.
