# Supplementary material for: Comparative Transcriptomic Analysis Reveals Domestication and Improvement Patterns of Broomcorn Millet (Panicum miliaceum L.)
Source: Int J Mol Sci. 2024 Oct 13;25(20):11012. doi: 10.3390/ijms252011012 (PMC11507134; doi:10.3390/ijms252011012)
Supplement: Supplementary file 1 [file ijms-25-11012-s001.zip › Table S1.pdf]

**Table S1.** Statistics of RNA-seq data of wild accessions, landraces, and improved cultivars of *Panicum miliaceum* L. at both seedling and filling stages used in this study. WS, LS, and IS indicate wild types, landraces, and improved cultivars at seedling stage, respectively; WF, LF, and IF present wild types, landraces, and improved cultivars at filling stage, respectively.

| **Group** | **Accession** | **Sample** | **Variety** | **Status** | **Stage** | **Number of clean reads** | **Q30 Percentage** | **Number of total reads** | **Number of mapped reads** |
|---|---|---|---|---|---|---|---|---|---|
| WS | WNX24 | WNX24S-1 | Yemizi | Wild accession | Seedling | 19,595,949 | 96.25% | 39,191,898 | 34,609,096 (88.31%) |
| | WNX24 | WNX24S-2 | Yemizi | Wild accession | Seedling | 21,286,665 | 96.01% | 42,573,330 | 38,214,875 (89.76%) |
| | WNX24 | WNX24S-3 | Yemizi | Wild accession | Seedling | 20,343,122 | 96.32% | 40,686,244 | 38,505,719 (94.64%) |
| | WNM8 | WNM8S-1 | Dianxingziganyemi | Wild accession | Seedling | 24,390,256 | 95.49% | 48,780,512 | 45,749,291 (93.79%) |
| | WNM8 | WNM8S-2 | Dianxingziganyemi | Wild accession | Seedling | 22,620,421 | 95.77% | 45,240,842 | 42,361,119 (93.63%) |
| | WNM8 | WNM8S-3 | Dianxingziganyemi | Wild accession | Seedling | 22,156,017 | 95.32% | 44,312,034 | 41,163,089 (92.89%) |
| WF | WNX24 | WNX24F-1 | Yemizi | Wild accession | Filling | 19,260,673 | 95.26% | 38,521,346 | 23,854,096 (61.92%) |
| | WNX24 | WNX24F-2 | Yemizi | Wild accession | Filling | 23,117,158 | 95.51% | 46,234,316 | 19,857,890 (42.95%) |
| | WNX24 | WNX24F-3 | Yemizi | Wild accession | Filling | 20,556,054 | 93.82% | 41,112,108 | 13,423,364 (32.65%) |
| | WNM8 | WNM8F-1 | Dianxingziganyemi | Wild accession | Filling | 21,897,232 | 93.67% | 43,794,464 | 18,868,300 (43.08%) |
| | WNM8 | WNM8F-2 | Dianxingziganyemi | Wild | Filling | 22,249,501 | 94.49% | 44,499,002 | 32,782,459 |

| | | | | | | | | | |
|---|---|---|---|---|---|---|---|---|---|
| | | | | accession | | | | | (73.67%) |
| | WNM8 | WNM8F-3 | Dianxingziganyemi | Wild accession | Filling | 24,526,433 | 94.23% | 49,052,866 | 28,867,704 (58.85%) |
| LS | LLN5 | LLN5S-1 | Dabaimizi | Landrace | Seedling | 25,803,592 | 94.21% | 51,607,184 | 42,582,691 (82.51%) |
| | LLN5 | LLN5S-2 | Dabaimizi | Landrace | Seedling | 23,471,436 | 94.14% | 46,942,872 | 41,565,026 (88.54%) |
| | LLN5 | LLN5S-3 | Dabaimizi | Landrace | Seedling | 23,720,105 | 94.32% | 47,440,210 | 41,108,455 (86.65%) |
| | LJL8 | LJL8S-1 | Yanpibao | landrace | Seedling | 19,649,000 | 94.13% | 39,298,000 | 37,483,707 (95.38%) |
| | LJL8 | LJL8S-2 | Yanpibao | landrace | Seedling | 22,588,955 | 94.14% | 45,177,910 | 42,145,692 (93.29%) |
| | LJL8 | LJL8S-3 | Yanpibao | landrace | Seedling | 23,018,865 | 93.81% | 46,037,730 | 41,886,553 (90.98%) |
| LF | LLN5 | LLN5F-1 | Dabaimizi | Landrace | Filling | 24,090,793 | 93.93% | 48,181,586 | 31,908,698 (66.23%) |
| | LLN5 | LLN5F-2 | Dabaimizi | Landrace | Filling | 22,337,861 | 93.62% | 44,675,722 | 38,038,431 (85.14%) |
| | LLN5 | LLN5F-3 | Dabaimizi | Landrace | Filling | 22,325,545 | 94.09% | 44,651,090 | 36,122,476 (80.90%) |
| | LJL8 | LJL8F-1 | Yanpibao | landrace | Filling | 19,166,201 | 93.55% | 38,332,402 | 17,929,180 (46.77%) |
| | LJL8 | LJL8F-2 | Yanpibao | landrace | Filling | 24,487,660 | 94.34% | 48,975,320 | 26,754,789 (54.63%) |
| | LJL8 | LJL8F-3 | Yanpibao | landrace | Filling | 22,781,127 | 94.13% | 45,562,254 | 27,846,293 |

| | | | | | | | | | |
|---|---|---|---|---|---|---|---|---|---|
| | | | | | | | | | (61.12%) |
| IS | ISX63 | ISX63S-1 | Yanshu 7 | Improved cultivars | Seedling | 20,590,292 | 93.67% | 41,180,584 | 37,656,732 (91.44%) |
| | ISX63 | ISX63S-2 | Yanshu 7 | Improved cultivars | Seedling | 19,976,524 | 93.75% | 39,953,048 | 37,492,759 (93.84%) |
| | ISX63 | ISX63S-3 | Yanshu 7 | Improved cultivars | Seedling | 21,609,111 | 93.57% | 43,218,222 | 40,261,737 (93.16%) |
| | IGS169 | IGS169S-1 | Longmi 5 | Improved cultivars | Seedling | 22,550,989 | 94.39% | 45,101,978 | 38,816,862 (86.06%) |
| | IGS169 | IGS169S-2 | Longmi 5 | Improved cultivars | Seedling | 19,902,956 | 93.49% | 39,805,912 | 34,748,759 (87.30%) |
| | IGS169 | IGS169S-3 | Longmi 5 | Improved cultivars | Seedling | 23,862,685 | 93.75% | 47,725,370 | 45,664,707 (95.68%) |
| IF | ISX63 | ISX63F-1 | Yanshu 7 | Improved cultivars | Filling | 21,633,010 | 96.55% | 43,266,020 | 38,648,566 (89.33%) |
| | ISX63 | ISX63F-2 | Yanshu 7 | Improved cultivars | Filling | 20,648,100 | 96.23% | 41,296,200 | 25,251,900 (61.15%) |
| | ISX63 | ISX63F-3 | Yanshu 7 | Improved cultivars | Filling | 22,057,698 | 94.15% | 44,115,396 | 33,846,327 (76.72%) |
| | IGS169 | IGS169F-1 | Longmi 5 | Improved cultivars | Filling | 24,320,702 | 94.39% | 48,641,404 | 29,544,763 (60.74%) |
| | IGS169 | IGS169F-2 | Longmi 5 | Improved cultivars | Filling | 20,694,771 | 93.40% | 41,389,542 | 14,262,061 (34.46%) |
| | IGS169 | IGS169F-3 | Longmi 5 | Improved cultivars | Filling | 22,582,242 | 94.30% | 45,164,484 | 28,536,218 (63.18%) |
